# Whole-Genome Phylogenetic Analysis Reveals a Wide Diversity of Non-O157 STEC Isolated From Ground Beef and Cattle Feces

**DOI:** 10.3389/fmicb.2020.622663

**Published:** 2021-01-18

**Authors:** Sebastián Gutiérrez, Leonela Díaz, Angélica Reyes-Jara, Xun Yang, Jianghong Meng, Narjol González-Escalona, Magaly Toro

**Affiliations:** ^1^Instituto de Nutrición y Tecnología de los Alimentos (INTA), Universidad de Chile, Macul, Santiago, Chile; ^2^Department of Nutrition and Food Science, University of Maryland, College Park, College Park, MD, United States; ^3^Joint Institute for Food Safety and Applied Nutrition, University of Maryland, College Park, College Park, MD, United States; ^4^U.S. Food and Drug Administration, Center for Food Safety and Applied Nutrition, College Park, MD, United States

**Keywords:** STEC, non-O157 *E. coli*, genomics, diversity, WGS

## Abstract

Shiga toxin-producing *Escherichia coli* (STEC) causes foodborne outbreaks that can lead to complications such as hemolytic uremic syndrome. Their main reservoir is cattle, and ground beef has been frequently associated with disease and outbreaks. In this study, we attempted to understand the genetic relationship among STEC isolated in Chile from different sources, their relationship to STEC from the rest of the world, and to identify molecular markers of Chilean STEC. We sequenced 62 STEC isolated in Chile using MiSeq Illumina. *In silico* typing was determined using tools of the Center Genomic Epidemiology, Denmark University (CGE/DTU). Genomes of our local STEC collection were compared with 113 STEC isolated worldwide through a core genome MLST (cgMLST) approach, and we also searched for distinct genes to be used as molecular markers of Chilean isolates. Genomes in our local collection were grouped based on serogroup and sequence type, and clusters were formed within local STEC. In the worldwide STEC analysis, Chilean STEC did not cluster with genomes of the rest of the world suggesting that they are not phylogenetically related to previously described STEC. The pangenome of our STEC collection was 11,650 genes, but we did not identify distinct molecular markers of local STEC. Our results showed that there may be local emerging STEC with unique features, nevertheless, no molecular markers were detected. Therefore, there might be elements such as a syntenic organization that might explain differential clustering detected between local and worldwide STEC.

## Introduction

Shiga toxin-producing *Escherichia coli* (STEC) is a significant pathogen; it can cause serious diseases in humans, not only as sporadic cases but also as outbreaks of foodborne disease (FAO/WHO, 2018). Cattle are the main STEC reservoir, and beef has been frequently associated with human disease, but STEC has been also isolated from other sources (Álvarez-Suárez et al., 2016; Sanches et al., 2017; Rios et al., 2019). The main STEC virulence factors are Shiga toxins, which are encoded by genes *stx*_1_ and *stx*_2_ and their subtypes. Shiga toxins are required for STEC pathogenicity and play a key role in complications such as hemorrhagic colitis and hemolytic uremic syndrome (HUS) (FAO/WHO, 2018). It is estimated that STEC causes over 2,800,000 cases/year worldwide and 3,890 cases of HUS. In Latin America, STEC is endemic and represents 2% of cases of acute diarrhea and up to 30% of bloody diarrhea, and Chile, Uruguay, and Argentina are the most affected countries in the area (Torres et al., 2018). STEC is considered an emergent pathogen in Chile; the main transmission route is through contaminated foods affecting mainly children between 6 months and 4 years, and mortality rates reach 3% (Vidal et al., 2010; ISP, 2017; Cavagnaro, 2019). Chilean law dictates that STEC is a pathogen under mandatory laboratory surveillance, so every clinical laboratory must send their isolates to the National Public Health laboratory (ISP) for confirmation. The serotypes most frequently causing disease in 2010–2016 were O157:H7 (55.7%), O26:H11 (28.5%), and O26:H- (6.4%) (ISP, 2017).

Shiga toxin-producing *E. coli* isolated from different sources have been characterized worldwide. STEC O157 has been the most studied serogroup, but other serotypes isolated from human clinical cases, animals, foods, and the environment have been also characterized. Reports indicate a wide variety of serotypes and sequence types of STEC isolated in Iran, Japan, Argentina, Brazil, among others, from diverse sources. Many of these studies have used techniques such as MLST, traditional serotyping, and PFGE to study STEC isolates (Feng et al., 2014; Cadona et al., 2016; Ferdous et al., 2016; Jajarmi et al., 2017; Nakamura et al., 2018).

Many countries currently study foodborne pathogens and investigate outbreaks by analysis of the Whole Genome Sequencing (WGS) of the isolates. This technique provides high discriminating power among isolates (Nadon et al., 2017; Rantsiou et al., 2018; Simon et al., 2018; Jenkins et al., 2019). A year after the implementation of WGS for food safety purposes in the United States, outbreaks were reported faster, and more cases were linked to an outbreak than before (Jagadeesan et al., 2019). WGS is also useful to characterize isolates and to analyze their phylogenetic relationship (Holmes et al., 2018). For example, 152 STEC serotype O26 from New Zealand were compared to STEC isolated in the rest of the world. Interestingly, all New Zealand isolates clustered together regardless of the Shiga toxin type they carried (Browne et al., 2019). Even when some STEC sequence types are distributed worldwide, this and other evidence using WGS approach suggests that STEC phylogeny is influenced by the origin of the geographic isolate and that there are highly conserved genes linked to local environments where they evolved (Yu et al., 2018; Browne et al., 2019). Therefore, there may be STEC molecular markers and distinct genes based on their geographic origin (Kiel et al., 2018).

An earlier study characterized the diversity of *E. coli* O157 from Chile obtained from diverse sources (human clinical cases, foods, and animals) (Ríos et al., 2009). This study demonstrated the diversity among O157 STEC and found 37 different PFGE profiles among 39 isolates. However, other STEC serotypes have not been studied. In the present study, we characterized non-O157 STEC isolated in Chile mainly from cattle feces and ground beef using genomic analysis and studied the relationship between these isolates and others collected worldwide. Also, considering the geographical barriers that protect Chile and the *E. coli* genome plasticity, we searched for distinct regional genetic markers of STEC isolated locally.

## Methodology

### Isolates and Whole-Genome Sequencing

We obtained 62 STEC from cattle (*n* = 31) and ground beef (*n* = 27) in Chile from 2016 to 2017 (Toro et al., 2018). Additionally, we sequenced two STEC isolates obtained from wild bird feces (isolated in 2015) and two from goat cheese (obtained in 2012) from our collection (Table 1). DNA extraction was performed with the DNeasy Blood and Tissue kit (Qiagen, United States) at the Laboratory of Microbiology and Probiotics, INTA, University of Chile. Library preparation and WGS were performed at the US FDA Center for Food Safety and Applied Nutrition (CFSAN) genomics laboratory. Libraries were prepared with the Nextera XT kit (Illumina, United States), and sequences were obtained with the MiSeq platform with the 250 pair-end reads, Illumina^®^.

**TABLE 1:** Isolates and genomic information of STEC obtained in Chile included in the study.

**Isolate**	**CFSAN**	**NCBI Accession**	**Isolation**	**Place of**	**Genome**	**Number of**	**Isolation**	**Sequence**	***In silico***	**Phylogroup**	***stx***	***eae***
**name**	**number**	**number**	**year**	**isolation**	**size**	**contigs**	**Source**	**Type**	**Serotype**		***gene***	***gene***
123-B-9	CFSAN066312	SAMN07446203	2016	RM	5,046,459	111	Beef	ST-297	O93:H46	B1	2c	
127-A-4	CFSAN066313	SAMN07446202	2016	RM	4,825,625	791	Beef	ST-2387	O185:H7	B1	2c	
128-A-4	CFSAN066314	SAMN07446201	2016	RM	4,877,087	406	Beef	ST-297	O93:H46	B1	2c	
135-A-8	CFSAN066316	SAMN07446199	2016	RM	4,957,902	133	Beef	ST-297	O93:H46	B1	2c	
139A-3	CFSAN066317	SAMN07446198	2016	RM	4,944,551	104	Beef	ST-2458	O91:H21	B1	2a	
186-7	CFSAN066396	SAMN07444439	2016	RM	5,014,161	249	Beef	ST-58	O116:H21	B1	2	
19-6	CFSAN066388	SAMN07444447	2016	LR	5,469,316	333	Cattle	ST-21	O26:H11	B1	1a	+
200A-3	CFSAN066319	SAMN07446196	2016	RM	4,962,598	93	Beef	ST-101	O82:H8	B1	1a	
201A-9	CFSAN066320	SAMN07446195	2016	RM	5,053,385	171	Beef	ST-101	O82:H8	B1	1a,2a	
210-2-1	CFSAN066399	SAMN07444436	2016	RM	5,146,150	108	Beef	ST-56	O113:H21	B1	2b/c	
232-A4*	**CFSAN066322**	**SAMN07446193**	**2016**	**RM**	**5,008,621**	**73**	**Beef**	**ST-2387**	**O185:H7**	**B1**	**2c**	
24-A-1	CFSAN066302	SAMN07446213	2016	RM	5,197,541	552	Beef	ST-1125	ONT:H19	B1	2a	
245-A8	CFSAN066323	SAMN07446192	2016	RM	5,115,199	309	Beef	ST-332	O171:H2	B1	2c	
260-A2	CFSAN066324	SAMN07446191	2016	RM	5,130,928	167	Beef	ST-297	O93:H46	B1	2	
283-A5	CFSAN066325	SAMN07446190	2016	RM	5,076,143	147	Beef	ST-2388	O115:H27	B1	2c	
2B-i	CFSAN066353	SAMN07446251	2016	LR	5,230,807	496	Cattle	ST-329	O3:H12	A	1a	
31-A-8	CFSAN066303	SAMN07446212	2016	RM	4,976,991	394	Beef	ST-2387	O185:H7	B1	2c	
314-A4	CFSAN066327	SAMN07446188	2016	RM	5,225,902	164	Beef	ST-677	O174:H21	B1	2c	
315-B8	CFSAN066328	SAMN07446187	2016	RM	5,131,154	164	Beef	UKN	O116:H21	B1	1a	
346-A1	CFSAN066331	SAMN07446184	2016	RM	5,063,522	540	Beef	ST-446	O22:H8	B1	2c	
366-A3	CFSAN066332	SAMN07446217	2016	RM	5,066,620	151	Beef	ST-223	O113:H21	B1	2a	
400-B10	CFSAN066334	SAMN07446280	2016	RM	4,959,045	143	Beef	UNK	O174:H28	B1	2	
46-B-8	CFSAN066305	SAMN07446210	2016	RM	5,032,256	361	Beef	ST-156	O174:H28	B1	2	
55-A-3	CFSAN066306	SAMN07446209	2016	RM	4,937,842	207	Beef	ST-446	O22:H8	B1	2c	
57B2-2	CFSAN066390	SAMN07444445	2016	LR	5,073,597	147	Cattle	ST-297	O130:H11	B1	2	
5A-3-2	CFSAN066301	SAMN07446214	2016	RM	5,037,021	308	Beef	ST-1613	ONT:H21	B1	1,2	
62-B-1	CFSAN066307	SAMN07446208	2016	RM	5,123,335	187	Beef	ST-677	O174:H21	B1	2c	
73-B2	CFSAN066378	SAMN07446222	2016	LR	5,069,564	640	Cattle	ST-332	O171:H2	B1	2c	
81-A-3	CFSAN066308	SAMN07446207	2016	RM	4,968,241	115	Beef	ST-297	O93:H46	B1	2c	
82-A-7	CFSAN066309	SAMN07446206	2016	RM	5,121,613	193	Beef	ST-677	O174:H21	B1	2c	
85-B1	CFSAN066379	SAMN07446221	2016	LR	5,155,936	448	Cattle	ST-718	O168:H8	B1	2g	
93-A8	CFSAN066380	SAMN07446220	2016	LR	5,037,525	125	Cattle	ST-223	O113:H21	B1	2a	
94-A4	CFSAN066381	SAMN07446219	2016	LR	5,031,961	121	Cattle	ST-718	O168:H8	B1	2	
97-A-5	CFSAN066310	SAMN07446205	2016	RM	4,937,858	703	Beef	ST-223	O113:H21	B1	2a	
A2-1	CFSAN066340	SAMN07446271	2016	LI	5,004,899	371	Cattle	ST-58	O116:H21	B1	2a	
A3-1	CFSAN066341	SAMN07446270	2016	LI	5,006,376	233	Cattle	ST-223	O113:H19	B1	1,2	
A4-VI	CFSAN066342	SAMN07446268	2016	LI	5,208,584	219	Cattle	ST-718	O168:H8	B1	2g	
D27-10	CFSAN066398	SAMN07444437	2015	AP	5,416,539	172	Wild bird feces	ST-675	O76:H19	B1	1c	
D27-8	CFSAN066397	SAMN07444438	2015	AP	4,899,164	427	Wild bird feces	ST-4392	O149:H8	B1	1a	
E6-4	CFSAN066346	SAMN07446262	2016	LI	5,104,668	219	Cattle	ST-660	O172:H25	A	2a	+
E6-III	CFSAN066345	SAMN07446263	2016	LI	5,374,188	255	Cattle	ST-306	O98:H21	B1	1a	+
E7-2	CFSAN066349	SAMN07446257	2016	LI	5,079,860	380	Cattle	ST-660	O172:H28	A	2a	+
H131	CFSAN066338	SAMN07446274	2009	RM	5,388,489	281	Goat cheese	ST-675	O76:H9	B1	1c	
H135	CFSAN066339	SAMN07446273	2009	RM	5,386,030	448	Goat cheese	ST-675	O76:H19	B1	1c	
M10-3	CFSAN066368	SAMN07446232	2016	LR	4,928,210	147	Cattle	ST-2458	O91:H21	B1	2a	
M15-3	CFSAN066370	SAMN07446230	2016	LR	5,078,930	154	Cattle	ST-443	O153/O178:H19	B1	1,2	
M2-3-1	CFSAN066391	SAMN07444444	2016	LR	4,943,708	339	Cattle	ST-58	O116:H21	B1	2a	
M21-1	CFSAN066371	SAMN07446229	2016	LR	5,036,324	204	Cattle	ST-58	O116:H21	B1	2	
M21-2	CFSAN066372	SAMN07446228	2016	LR	5,059,469	182	Cattle	ST-58	O116:H21	B1	2	
M22-1	CFSAN066373	SAMN07446227	2016	LR	5,093,813	232	Cattle	ST-297	O93:H46	B1	2	
M29-4	CFSAN066375	SAMN07446225	2016	LR	5,044,200	528	Cattle	ST-443	O153/O178:H19	B1	2c	
M4-1	CFSAN066365	SAMN07446235	2016	LR	5,143,415	177	Cattle	ST-192	O153/O178:H19	B1	2	
M41-7	CFSAN066376	SAMN07446224	2016	LR	4,964,719	506	Cattle	ST-58	O116:H21	B1	2	
M9-3	CFSAN066366	SAMN07446234	2016	LR	5,043,027	111	Cattle	ST-657	O183:H18	F	1,2	
P2-2-8	CFSAN066354	SAMN07446250	2016	LI	5,009,832	291	Cattle	ST-173	O181:H49	B1	2c	
P3-5-5	CFSAN066355	SAMN07446249	2016	LI	5,256,628	157	Cattle	ST-718	O168:H8	B1	2g	
P37-1	CFSAN066386	SAMN07444449	2016	LI	5,215,899	195	Cattle	ST-443	O153/O178:H19	B1	1a,2a	
P4-1	CFSAN066382	SAMN07446218	2016	LI	4,912,075	416	Cattle	ST-223	O113:H21	B1	1a,2a	
p4-2-10	CFSAN066356	SAMN07446247	2016	LI	5,343,343	387	Cattle	ST-297	ONT:H8	B1	2	
p5-3-10	CFSAN066357	SAMN07446245	2016	LI	5,100,014	377	Cattle	ST-332	O171:H2	B1	2a	
P6-2-1	CFSAN066358	SAMN07446243	2016	LI	5,114,957	160	Cattle	ST-442	O91:H21	B1	2a	
P6-3-7	CFSAN066360	SAMN07446240	2016	LI	5,037,620	136	Cattle	ST-446	O22:H8	B1	2c	

*Genome used a template for the cgMLST analysis of our local collection. Place of isolation: Central Chile regions: RM (Santiago Metropolitan Region); LI (Libertador Bernardo O’Higgins Region). Southern Chile region: LR (Los Rios Region).

### Data Analysis and Genomic Characterization

Genomes were assembled using the CLC Genomics workbench platform version 7.6.1. with default parameters (Qiagen, United States), defining a minimum contig size of 500 bp. Isolates were typed using genomic information with the tools of the Center for Genomic Epidemiology (CGE)^1^. *In silico* serotyping were predicted with SeroTypeFinder2.0^2^ that uses target genes linked to the O somatic antigen (*wzx*, *wzy*, *wzm*, and *wzt*) and genes that define the H flagellar antigen (*fliC*, *flkA*, *fllA*, *flmA*, and *flnA*) (Joensen et al., 2015). An identity parameter (% ID) of 85% was selected, which corresponds to the minimum percentage of nucleotides that are identical among the genes in the database involved in the determination of serotypes. The minimum length selected was 60%, which corresponds to the percentage by which a sequence must overlap with a serotype gene to count as a hit. The determination of the allelic profiles or Sequence Type was performed through Multi Locus Sequence Typing version 2.0^3^, using the following housekeeping genes as reference: *adk*, *fumC*, *gyrB*, *icd*, *mdh*, *recA*, and *purA* (Larsen et al., 2012).

### Phylogenetic Analyses

#### Genomic Diversity of STEC Isolated in Chile

A phylogenetic reconstruction was performed with 62 Chilean STEC genomes (Table 1) based on a core genome MLST protocol (cgMLST) defined with Ridom SeqSphere v4.1.9 (Ridom GmbH, Germany). This approach uses an annotated genome as a template and defines targets, and then compares all the genomes to define the presence and absence of genes and allelic variations. Then, phylogenetic relationships among the genomes are calculated (Supplementary Table 1). Minimum spanning trees were used for data visualization, also generated with Ridom SeqSphere. We selected as a template genome *E. coli* K-12 (GenBank Accession, version: NC_000913.3) since it is the reference strain for the species, and it carries genes that characterize all *E. coli* despite their serotype. Clusters (highly related genomes) were defined as genomes with 10 or fewer allele differences. A whole-genome SNP phylogeny was used for a second phylogenetic study. Genomes were aligned using CSI phylogeny v1.4^4^ provided by CGE. *E. coli* K-12 was used as a reference (NC_000913.3), and default parameters were used for the analysis (Kaas et al., 2014). Once SNP were identified, a dendrogram was generated and calculated by the maximum likelihood method using the GTR + CAT model with 1000 bootstrap replicas in Fastreev2.1 in GalaxyTrakr version (Price et al., 2009). In parallel, we defined *in silico* phylogroups for each genome with the ClermonTyping v1.4 tool^5^ (Beghain et al., 2018). Finally, a dendrogram was visualized and edited with Evolview v2.0^6^ (He et al., 2016).

#### Comparison Between Chilean and Worldwide STEC

We performed a cgMLST study with Ridom SeqSphere using the 62 Chilean genomes (our local collection) and 113 whole STEC genomes worldwide carefully selected to represent each continent and serotypes present in our collection in order to increase the chances of clustering. Databases used were PATRIC^7^ and NCBI Sequence Archives (SRA)^8^. Genomes selected were classified into the following groups: Europe (*n* = 19), North America (*n* = 30), Asia (*n* = 17), Africa (*n* = 10), Oceania (*n* = 10), and South America (*n* = 27), isolated from human, domestic ruminants and food since 2001 to 2018, except for two genomes obtained in Europe in 1986 and 1993 (Supplementary Table 2). Genomes of STEC O113:H21 (*n* = 5) and STEC O116:H21 (*n* = 7) from these databases were intentionally added to the analysis because these serotypes were the most frequently found among our genomes (Supplementary Table 2). Clusters were also defined as genomes with fewer than 10 gene differences. Genomes from our local collection were uploaded to NCBI and visualized in the Pathogen Detection database^9^ which clusters related genomes with less than 50 SNP differences (NCBI). Over 80.000 *E. coli* and *Shigella* spp. genomes were in the Pathogen Detection database at the analysis date and were compared to our genomes (May 28, 2020).

### Identification of Molecular Markers for Chilean STEC

Two approaches were used to identify potential molecular markers in the Chilean genomes:

#### Approach Using a cgMLST Strategy

To perform this strategy, we first defined the core genome of our local collection. For this, we first defined our local template genome by selecting the one with the best assembly parameters (contig number and nucleotide number: Table 1) and annotated it in Prokka v1.13 (Seemann, 2014). Then, we created a project in Ridom SeqSphere+ to define the core genome of our local collection by comparing the 61 remaining local genomes to our local template genome; this procedure created a first list that contained core genes shared by all the STEC genomes in our local (Chilean) collection. Secondly, to identify potential molecular markers unique to our collection, we compared genes present in our local template against genes present in the *E. coli* K-12 genome. The latter represents the reference genome of all *E. coli*, including pathogens and non-pathogen strains, and it includes the genomic backbone of every *E. coli* which had to be discarded to find local markers. This created a second list of genes present only in our local template but not in *E. coli* K-12. Finally, to select candidate genes, we compared both lists: core genes of our local STEC collection versus those present only in the template but not in *E. coli* K-12. In this way we identified genes that were present in all STEC in our collection but not in *E. coli* K-12. Once all those genes were identified, their nucleotide sequences were screened in the NCBI database using BLASTn (Altschul et al., 1990). A potential marker was a gene that had less than 80% identity with any other sequence in the database (Kiel et al., 2018).

#### Approach Using a Pangenome Strategy

First, all 175 genomes in this study were annotated by Prokka v1.13 (Seemann, 2014). The pangenome was defined using the tool get_homologues, an open-source software package designed for the pangenomic and comparative-genomic analysis of bacterial strains (Contreras-Moreira and Vinuesa, 2013). The tool uses the scripts *./get_homologues.pl* and *./compare_clusters.pl* to detect ortholog genes through BLAST with the OrthoMCL (OMLC) and Bidirectional Best Hit (BDBH) algorithms, and to remove repeated genes. As a result, a presence/absence matrix is built for each gene/genome combination. Finally, with the script.*/parse_pangenome_matrix.pl*, the local collection pangenome (local pangenome list) is filtered against the worldwide pangenome (worldwide pangenome list) in order to define those genes present in the local collection but not in STEC from other locations.

## Results

### Serotyping and Sequence Types of Chilean STEC

We identified 28 serotypes among the 62 Chilean genomes. The serotypes most frequently found were: O116:H21 (11.3%; 7/62), O93:H46 (9.7%; 6/62) and O113:H21 (8.1%; 5/62) (Table 1). SeroTypeFinder 2.0 did not identify the somatic antigen (O) for two genomes (3.2%), but only their flagellar antigen (genome 24-A-1 serotype ONT:H19 and 5A-3-2 serotype ONT:H21) (Table 1). This might be due to coverage issues in the region implicated in O antigen determination (Lindsey et al., 2016). Also, this approach does not discriminate between serogroups O153 and O178. As a result, 4/62 genomes (6.5%) were designated as O153:H19 or O178:H19 (O153/O178:H19). Multi-locus Sequence Typing (MLSTv2.0) indicated 26 different ST; the most frequently reported were ST297 and ST58 (9.7%; 6/62 each), followed by ST223 (8.1%; 5/62) and ST718 (6.5%; 4/62) (Table 1). Two new allele profiles were found (3.2%) in genomes 315-B8 and 400-B10. Most genomes with the same sequence type were of the same serotype except genomes of ST223; four of these were serotype O113:H21 and one was O113:H19 (Table 1).

### Genomic Diversity of STEC Isolated in Chile

**FIGURE 1:**
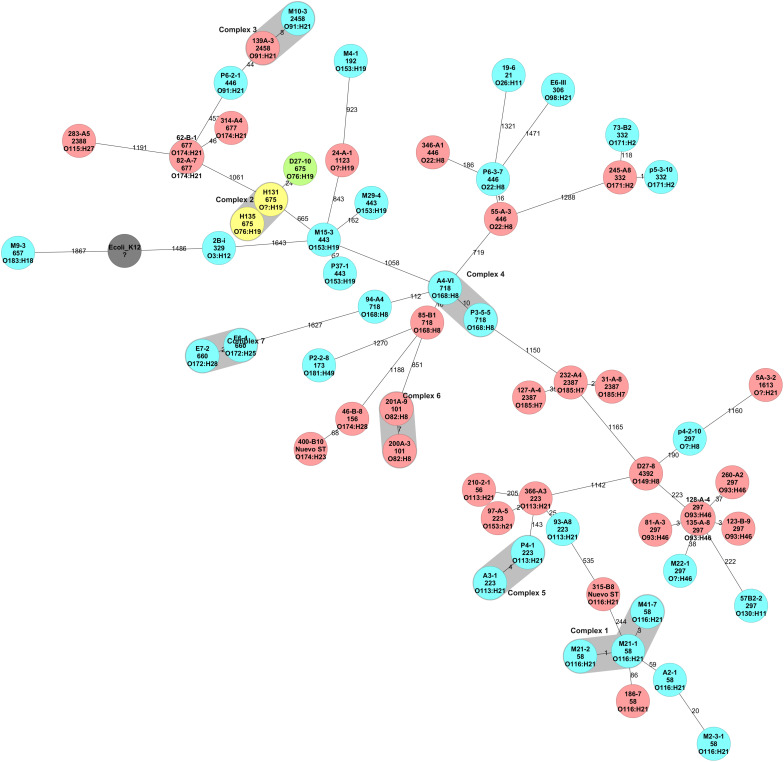
Minimum Spanning Tree of STEC isolated in Chile inferred from the cgMLST analysis. Reference genome was *E. coli* K-12 (Accession number NC_000913.3). The STEC core genome inclueded 1,974 genes. The number on the branches represent the allele difference between isolates. Clusters were defined as genomes with fewer than 10 allele differences and are identified as blue colors surrounding a group of genomes. Colors indicate isolation source: yellow, red, etc.

The core genome of our STEC collection was composed of 1,974 genes (Supplementary Table 1). The cgMLST showed that genomes were grouped based on serotype and sequence type. Out of the 62 genomes, 15 grouped into seven clusters while the remaining genomes did not group in a cluster. Four clusters included only STEC obtained from cattle stool, one cluster included both isolates obtained from goat cheese, and a single cluster had genomes of isolates of different origin–M10-3 from cattle feces and 139-A3 from ground beef (Figure 1).

**FIGURE 2:**
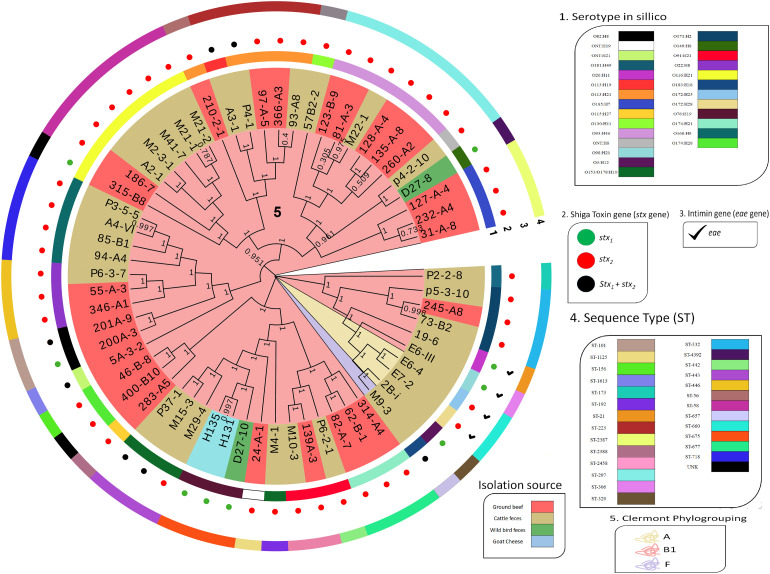
Dendrogram of 62 STEC isolated in Chile. Dendrogram calculated by the maximum likelihood method using the GTR + CAT model with 1000 bootstrap repetitions. The perimeter line color indicates serotype, *stx* gene, *eae* presence, and Sequence Type. Bootstrap value is indicated over each branch. Background color on branches indicates phylogroups.

The SNP analysis identified 86,739 SNPs among the STEC in the collection. A maximum-likelihood phylogeny reconstruction showed that genomes were grouped based on phylogroup and sequence type, regardless of their isolation source (Figure 2). STEC from phylogroups A (*n* = 3) and F (*n* = 1) were obtained from cattle stool while phylogroup B1 genomes were obtained from all four sources in the study (Figure 2).

### Diversity of a Collection of Chilean STEC Associated With Worldwide Isolates

**FIGURE 3:**
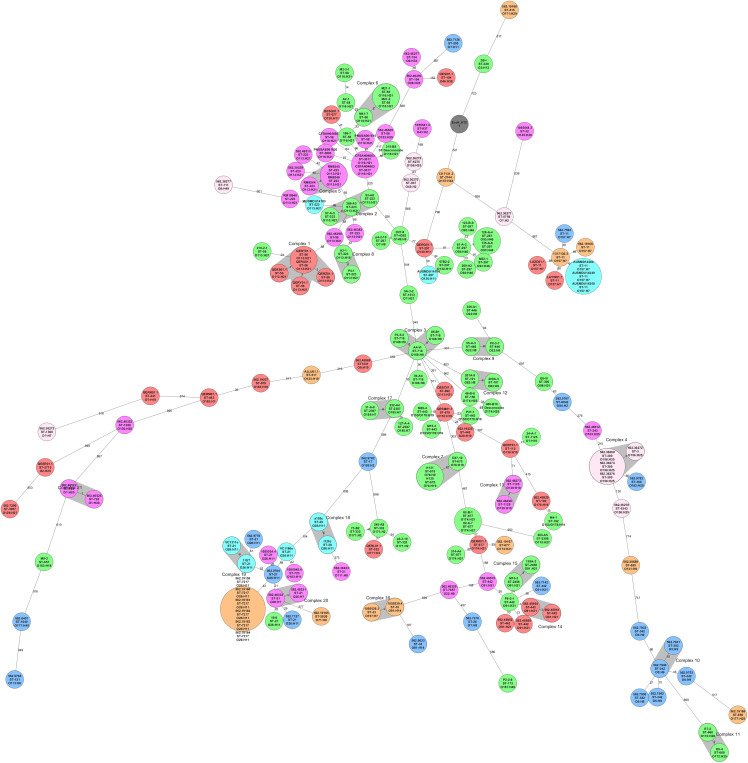
Minimum Spanning Tree of STEC isolated from different locations in the world (*n* = 113) and STEC isolated in Chile (*n* = 62) inferred from the cgMLST analysis. Reference genome was *E. coli* K-12 (Number accession NC_000913.3) and the clusters (highly related genomes) were defined as genomes with 10 or fewer allele differences. The size of each circle depends on the number of genomes determined as clones. All the genomes were grouped based on serotype and sequence type. Local STEC genomes (in green) were not closely related to 113 genomes isolated from other countries and grouped at the center of the figure, except by isolates in cluster 11 (E7-2 and E6-4) and isolate P2-2-8, all at the bottom of the figure.

The core genome of the 113 worldwide collection (Supplementary Table 2) and 62 genomes of local STEC included 1,018 genes. The minimum spanning tree showed that STEC genomes grouped based on serotype and sequence type. However, Chilean STEC grouped in the center of the Minimum Spanning Tree (MST). The exception was three isolates located in the out branches; cluster 11 included genomes E7-2 and E6-4 (serogroup O172), and the closest Chilean genome was 807 alleles deference, while genome M9-3 (serotype O181:H49) located 953 alleles away from the closest Chilean genome (A4-VI) (Figure 3). We identified 7 clusters including 15 Chilean STEC genomes (15/62), but they were not closely related to STEC isolated elsewhere (Figure 3). The NCBI Pathogen Detection platform indicated that the 62 Chilean STEC of our collection did not cluster with any STEC reported to date (May 28, 2020; Supplementary Table 3). Nevertheless, this is based on a small sample size and could change as more strains are sequenced and added to the database.

### Detection of Molecular Markers in Chilean STEC

#### Approach Using a cgMLST Strategy

To define gene targets, genome 232-A4 was defined as a template since it reached the best quality parameters for assembly in the collection (5.01 Mb and 73 contigs); finally, 4,886 genes were identified in this genome (Table 1). The core genome of the Chilean STEC collection included 3,166 genes, while the number of non-shared genes between 232-A4 and *E. coli* K12 was 1,001. Only 23 genes were present in both lists, representing genes exclusively present in the Chilean STEC genomes. BLAST informed that most of these potential genetic markers encode transporters, CRISPR regions, and transcription regulators in different bacterial species of the family *Enterobacteriaceae* such as *E. coli*, *Salmonella enterica*, *Escherichia albertii*, and *Shigella* spp. Two of these genes encoded hypothetical proteins or non-characterized proteins, however, both genes had been previously described and are distributed in *E. coli* complete genomes from around the world with identities of 100% and *e*-values close to 0 (Supplementary Table 4).

#### Approach Using a Pangenome Strategy

**FIGURE 4:**
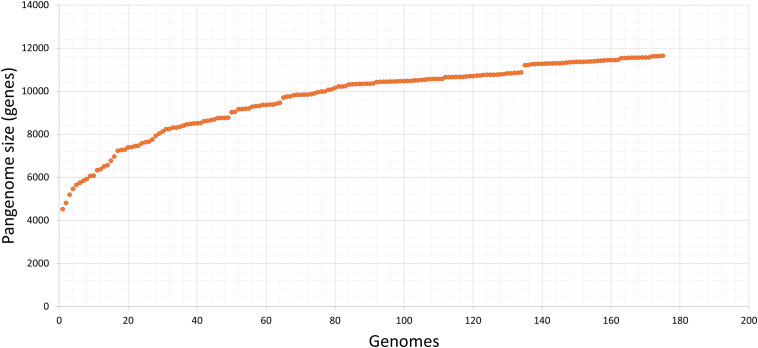
Pangenome of our collection of STEC isolated in Chile. Input data was generated with get_homologues, and visualization with graph tool of Microsoft, excel. The estimated pangenome was 11,650 genes. Each point corresponds to one genome; after the consecutive analysis of each one, the number of estimated genes of the STEC pangenome increases.

All 175 annotated genomes created a pangenome of 11,650 genes (Figure 4). The pangenome matrix and the comparison among genes did not identify any gene present exclusively in the Chilean pangenome, thus we did not detect any potential marker using this strategy.

## Discussion

Shiga toxin-producing *E. coli* characterization is relevant to improve epidemiological surveillance and for source attribution of foodborne infections. In the past, serotyping (traditional and molecular) and sequence typing provided relevant information about STEC epidemiology, and it helped to attribute disease and foodborne outbreaks to certain STEC serotypes and ST (Riley, 2014; Ferdous et al., 2016). Whole-genome sequencing has arisen recently as a crucial methodology that improves isolate characterization and outbreak investigation (Simon et al., 2018; Jenkins et al., 2019). To describe genomes of STEC circulating in Chile better, and to identify their relationship with genomes in the rest of the world, we sequenced the complete genome of 62 Chilean STEC. We observed high diversity of STEC obtained in Chile from two main sources: ground beef and cattle stool (Table 1 and Figures 1, 2). Chilean STEC genomes (*n* = 62) were of 28 serotypes (Table 1). Similar findings have been reported in The Netherlands, where 42 different serotypes were identified in 406 STEC genomes, and serotype and isolation location were unrelated (Ferdous et al., 2016). In Argentina, 47 serotypes were described among 153 STEC isolates (Blanco et al., 2004). In both cases, several of the serotypes had been reported as causing human disease (Blanco et al., 2004; Ferdous et al., 2016).

The most frequently reported serotypes in this study were O116:H21, O93:H46, and O113:H21, obtained from ground beef and cattle feces. STEC O113:H21 has been isolated in the United States and Canada from cattle and swine feces, as well as from water sources surrounding these animal farms (Quiñones et al., 2017). This serotype has been reported as causing HUS in both countries and also in Australia, but North American and Australian cases occurred over 11 years ago (Paton et al., 1999; Mellmann et al., 2008; Käppeli et al., 2011). Studies in Argentina showed that STEC O113:H21 has been isolated also from beef and cattle, and it has been recently isolated from humans, pointing out that this is an emerging serotype in the country (DebRoy et al., 2004; Cadona et al., 2016; Sanso et al., 2018). Studies in Brazil have also isolated this serotype from cattle (Bando et al., 2017; Dos Santos et al., 2018). Official reports in Chile identified serogroup O113 as one of the most frequently isolated in beef (ISP, 2017). These results indicate that STEC O113:H21 is a serotype circulating among these countries and that it is causing human diseases. This could be explained by the geographical closeness and the extensive meat trade among these countries.

We report a single isolate of the big six group defined by the USDA and FDA (Brooks et al., 2005); an O26:H11 isolate obtained from cattle feces. This serotype is very relevant because it has caused multiple outbreaks in humans, especially in the United States (Hines et al., 2017; Scavia et al., 2018). The national institute for public health in Chile (ISP) reported that after O157:H7, O26:H11 is the most frequently reported serotype as a cause of STEC disease in the country (ISP, 2017). The last official STEC report in Chile indicates that the most frequently isolated serotypes from beef were O76, O113, O116, and O22 (ISP, 2017). All these serogroups were found in our collection (Table 1). This highlights the importance of having a better understanding of potentially pathogenic STEC isolated from foods and their relationship to human clinical disease. Epidemiological surveillance in the whole food production chain should be improved by the institutions in charge of public health in all countries.

Phylogenetic analysis using WGS provides a greater resolution that helps to determine relatedness among isolates. This type of analysis allows automated and more robust epidemiological surveillance (Inns et al., 2017; Holmes et al., 2018; Jagadeesan et al., 2019). In this study, phylogenetic analysis of the 62 Chilean isolates using cgMLST defined a core genome of 1,974 genes. However, it is important to note that core genomes are highly influenced by the reference genome selected as well as the closeness between isolates being analyzed. In this regard, we selected the genome of *E. coli* K-12 (NC_000913.3) as a reference genome for comparing strains. Recent studies suggested that *E. coli* of phylogroups B2, D, F, and G could be more ancestral than genomes phylogroup A, such as *E. coli* K-12 (Gonzalez-Alba et al., 2019). Therefore, the core genome defined in this study might change when choosing a more ancestral genome as a reference. In our analysis, genomes were grouped based on their allelic profile and serotype. Similar results have been reported by researchers in The Netherlands where a core genome of 132 STEC isolates included 2,069 genes; they also grouped based on ST (Ferdous et al., 2016). In our study, we defined cluster complexes among genomes with fewer than 10 gene differences, but this not necessarily means that those isolates were closely related. In this study, our STEC genomes only formed seven clusters. Only one cluster was formed by isolates of different origin; isolate M10-3 from cattle feces from southern Chile and 139A-3 from ground beef isolated in central Chile formed this cluster (Figure 1). These genomes only displayed eight allele differences (out of 1,974 core genes), and both were serotype O91:H21 and ST 2458; however, a cgMLST including only the three isolates of this serotype confirmed that these isolates were not clonal (Supplementary Figures 1A,B). Serotype O91:H11 has been isolated from various sources such as milk, ground meat, and cattle feces in various parts of the world, and it is described among LEE negative isolates that have caused cases of HUS in humans (Pradel et al., 2008; Madic et al., 2009; Mellmann et al., 2009; Galli et al., 2010). Additionally, ST 2458 has been associated with Latin America more frequently than with Europe or North America (Feng et al., 2017).

Our collection of Chilean STEC genomes was not closely related to 113 genomes isolated from other countries. Even among selected genomes of the same serotypes (O113:H21 and O116:H21, the most frequently found serotypes in our collection), no clusters were formed. NCBI’s Pathogen Detection analyses and rapidly compares foodborne pathogenic genomes from foods, animals, and human patients from all around the world; it can find closely related isolates, helping to improve public health surveillance. We tracked our genomes in the Pathogen Detection platform which had over 80.000 *E. coli* genomes available for comparison at the date of analysis, but no close relationship between our 62 genomes and other genomes was found. Pathogen Detection comparison uses a whole-genome SNP approach, and highly related isolates are separated by less than 50 SNPs. This result may indicate that STEC circulating in Chile have some unique characteristics linked to our country.

Recent literature indicates that the genomic content of STEC is strongly influenced by the isolation location, and *E. coli* genomic plasticity would allow the evolution of a STEC population in a defined region (Browne et al., 2019). This and the results described above led us to hypothesize that it might be possible to identify molecular markers of STEC isolated in Chile. We used two different approaches in the search for markers; however, we failed to identify genetic markers limited to Chile. Although we found 23 genes that were possible candidates, all of them had been previously documented in different *E. coli* pathotypes and even in other Enterobacteriaceae. This result might have been due to the use of a genome as a template to identify genes, restricting the analysis to those present in that particular genome. Therefore, we tested a novel, more comprehensive approach: pangenome comparison. This approach does not use reference genomes to define genes. Instead, it annotates all genomes in the collection before comparing them. Despite the effort, we did not detect any markers. Similar results were obtained in Germany, where a research group analyzed 254 STEC genomes of different serotypes to identify new molecular markers besides *stx*_1_ and *stx*_2_ and attributed the failure to the high plasticity of STEC genomes and STEC diversity (Kiel et al., 2018). Since STEC from Chile were not related to genomes from the rest of the world, we believe that there may be unique characteristics that allow STEC genomes to be differentiated geographically. We hypothesize that the marker might not be a single gene, but a specific synteny could be a marker, and that the approach used was not able to detect the organization.

The present study demonstrated that STEC from Chile are diverse, and they are not closely related to STEC from the rest of the world, indicating that new, undescribed lineages might be emerging in the area.

## Data Availability Statement

The datasets presented in this study can be found in online repositories. The names of the repository/repositories and accession number(s) can be found below: https://www.ncbi.nlm.nih.gov/, SAMN07 444436 SAMN07 444437 SAMN07 444438 SAMN07 444439 SAMN07 444444 SAMN07 444445 SAMN07 444447 SAMN07 444449 SAMN07 446184 SAMN07 446187 SAMN07 446188 SAMN07 446190 SAMN07 446191 SAMN07 446192 SAMN07 446193 SAMN07 446195 SAMN07 446196 SAMN07 446198 SAMN07 446199 SAMN07 446201 SAMN07 446202 SAMN07 446203 SAMN07 446205 SAMN07 446206 SAMN07 446207 SAMN07 446208 SAMN07 446209 SAMN07 446210 SAMN07 446212 SAMN07 446213 SAMN07 446214 SAMN07 446217 SAMN07 446218 SAMN07 446219 SAMN07 446220 SAMN07 446221 SAMN07 446222 SAMN07 446224 SAMN07 446225 SAMN07 446227 SAMN07 446228 SAMN07 446229 SAMN07 446230 SAMN07 446232 SAMN07 446234 SAMN07 446235 SAMN07 446240 SAMN07 446243 SAMN07 446245 SAMN07 446247 SAMN07 446249 SAMN07 446250 SAMN07 446251 SAMN07 446257 SAMN07 446262 SAMN07 446263 SAMN07 446268 SAMN07 446270 SAMN07 446271 SAMN07 446273 SAMN07 446274 SAMN07 446280.

## Ethics Statement

The animal study was reviewed and approved by Faculty of Veterinary Sciences of the University of Chile’s Ethics Committee. Written informed consent for participation was not obtained from the owners because the veterinarian in charge of sampling was the treating veterinarian at the farms, and he obtained oral consent from owners.

## Author Contributions

SG analyzed data and wrote the manuscript. LD and XY performed laboratory experiments. AR-J, JM, and NG-E provided materials and critically reviewed the manuscript. MT conceived the study, performed the data analysis, and wrote the manuscript. All authors contributed to the article and approved the submitted version.

## Conflict of Interest

The authors declare that the research was conducted in the absence of any commercial or financial relationships that could be construed as a potential conflict of interest.

## Footnotes

1 http://www.genomicepidemiology.org/

2 https://cge.cbs.dtu.dk/services/SerotypeFinder/

3 https://cge.cbs.dtu.dk/services/MLST/

4 https://cge.cbs.dtu.dk/services/CSIPhylogeny/

5 http://clermontyping.iame-research.center/

6 https://www.evolgenius.info//evolview/

7 https://www.patricbrc.org/

8 https://www.ncbi.nlm.nih.gov/sra

9 https://www.ncbi.nlm.nih.gov/pathogens

## Supplementary Material

Supplementary Table 1 Core genome of local STEC collection (*n* = 62).

Supplementary Table 2 Genomes used for the comparison between local STEC genomes and worldwide STEC genomes.

Supplementary Table 3 Chilean genomes and SNP to the closest genomes in the pathogen detection database (May 28, 2020).

Supplementary Table 4 NCBI Blast matches for 23 candidate genes to become genetic markers.
